# VEGF Expression, Cellular Infiltration, and Intratumoral Collagen Levels after Electroporation-Based Treatment of Dogs with Cutaneous Squamous Cell Carcinoma

**DOI:** 10.3390/life11121321

**Published:** 2021-11-30

**Authors:** Denner Dos Anjos, Cynthia Bueno, Ewaldo Mattos-Junior, Andrigo Barboza De Nardi, Carlos Eduardo Fonseca-Alves

**Affiliations:** 1 Department of Veterinary Clinic and Surgery, São Paulo State University, Jaboticabal 14884-900, Brazil; cynthiambueno@gmail.com (C.B.); andrigo@fcav.unesp.br (A.B.D.N.); 2 Department of Veterinary Surgery and Animal Anesthesiology, University of Franca, Franca 14404-600, Brazil; ewaldo.junior@unifran.edu.br; 3 Department of Veterinary Surgery and Animal Anesthesiology, São Paulo State University—UNESP, Botucatu 18618-681, Brazil; 4 Institute of Health Sciences, Bauru Campus, Paulista University—UNIP, Bauru 17048-290, Brazil

**Keywords:** cutaneous carcinoma, electroporation-based treatment, electrochemotherapy, bleomycin, doxorubicin, Masson’s trichrome

## Abstract

Canine cutaneous squamous cell carcinoma (SCC) is the most common type of skin cancer in tropical countries and is generally associated with exposure to solar ultraviolet light. It has a low metastatic rate, and local treatments, such as electrochemotherapy (ECT), promote long-term control or even complete remission. This study aimed to evaluate pre- and post-ECT treatment expression levels of vascular endothelial growth factor (VEGF) and CD31, cellular infiltration, and intratumoral collagen levels in dogs with cutaneous SCC. A prospective nonrandomized clinical study was performed using dogs with spontaneous SCC treated with ECT. Eighteen lesions from 11 dogs were included in the study. The expression levels of VEGF and CD31; cellular infiltration; and intratumoral collagen levels, as determined by Masson’s trichrome staining, were not significantly different from pre-treatment measurements on day 21 (*p* > 0.05). However, among cellular infiltration, the mixed subtype was correlated with better overall survival time when compared to lymphoplasmacytic and neutrophilic infiltration (*p* < 0.05). In conclusion, ECT had no effect on VEGF expression, cellular infiltration, or intratumoral collagen levels in dogs with cutaneous SCC at the time of evaluation, suggesting that early and late post-ECT-treatment phases should be considered.

## 1. Introduction

Squamous cell carcinoma (SCC) is a malignant epidermal keratinocyte tumor of the skin [1]. Its prevalence depends on geographic location. It is the most common type of skin cancer in tropical countries and is generally associated with solar ultraviolet light exposure levels ranging from 6.0% to 44.9% [2,3,4]. In addition to chronic sun exposure, a lack of pigment within the epidermis and sparse hair coverage are also risk factors for SCC development [5]. In both humans and animals, the prevalence of this tumor subtype increases with age, and there is no gender or racial predilection [6,7,8,9]. Previous studies have shown that actinic keratosis is an SCC precursor lesion; therefore, these lesions are common in humans and animals [10,11,12].

Similar to cutaneous SCC in humans, in dogs, it is usually locally invasive with low metastasis occurring late in the course of the disease, mainly to regional lymph nodes [13,14,15]. Due to its low metastasis risk, local treatments, such as surgery, cryosurgery, radiotherapy, and electrochemotherapy (ECT) are often able to provide local control, especially when initiated in the early stages of the disease and extends the survival time in most cases [16,17,18,19].

Anatomically, the epidermis is an avascular layer composed of keratinocytes, while the dermis is a fibroblast-rich network of collagen, capillaries, and lymphatic vessels that serve as an entrance for immune cells [20]. The skin serves as a physical and chemical barrier against harmful environmental agents, such as ultraviolet radiation. However, chronic exposure to this stress factor may lead to the initiation, promotion, and progression of skin cancer [21]. Thus, skin cancer is a global public health concern. SCC shows significant potential for recurrence, depending on the tumor size, lesion depth, perineural invasion, and immune system of the patient [22]. Skin contains a network of immune cell populations, such as skin-associated lymphoid tissue, residing both in the epidermis and the dermis [23]. Therefore, many treatment strategies have been used in immunotherapy for different types of skin cancers [24]. Despite the common occurrence of these neoplasms in dogs, little is known about the inflammatory response in skin tumors, as most studies have focused on mammary tumors in female dogs [25,26]. Understanding the complex biology of cSCC, including the characterization of the inflammatory response, may benefit future studies aimed at developing oncological immunotherapies. 

The extracellular matrix (ECM) is a complex microenvironment capable of inhibiting or stimulating cancer cells. Previous studies have shown that associations between chemicals and biological components, such as the blood vessels, ECM, vascular endothelial growth factor (VEGF), stromal cells, and leukocytes, may contribute to the cancer microenvironment [27,28,29,30,31].

Masson’s trichrome (MT) staining is a low-cost, practical method used for assessing the ECM, which is a major component of the tumor microenvironment. High intratumoral collagen levels have been associated with increased survival time in dogs with low-grade cutaneous mast cell tumors [29,32]. Compounds that inhibit the adhesion of tumor cells to the ECM have also been shown to prevent metastasis [33]. Aggressive carcinomas with shorter collagen fibers are present in female dogs with mammary tumors, which directly affects survival time [34]. This has also been observed in feline mammary tumors, wherein denser collagen, thicker and straighter fibers, and less identifiable tumor-stromal boundaries are associated with poorer outcomes, independent of clinical variables, thus increasing the predictive power of the clinical model [35]. 

ECT is an emerging non-ablative local treatment with cytotoxic and anti-vascular effects [36]. It is routinely used to treat cutaneous and subcutaneous tumors, including skin metastases of head and neck neoplasms. Due to its low toxicity, high safety, and high effectiveness, it is used for neoadjuvant or adjuvant treatment in animals and humans [36,37,38,39]. 

Few studies have evaluated the histopathological features before and after electroporation-based treatment [13,40]. Histopathological findings in colorectal liver metastases after ECT (8 weeks) show the presence of a band of fibrous tissue and chronic inflammatory infiltrates consisting of lymphocytes and plasma cells in the ablated area [41].

ECT also has vascular effects, including blood flow alterations, such as transient hypoperfusion; apoptosis of tumor endothelial cells; and the generation of a hypoxic environment [42,43]. Furthermore, no changes in collagen fibers have been reported in the treated zone 24 h after non-thermal irreversible electroporation, indicating that peri-cellular collagen (extracellular matrix) remains intact [44]. 

To the best of our knowledge, this is the first study to evaluate VEGF expression levels, cellular infiltration, and intratumoral collagen levels in tissue samples after ECT. To investigate the pathways of ECT action, we aimed to evaluate these parameters before (D0) and after (D21) electroporation-based treatment of dogs with cutaneous squamous cell carcinoma to determine whether they change with treatment. 

## 2. Materials and Methods

This study was performed in accordance with the National and International Recommendations for the Care and Use of Animals (National Research Council) [45] and was approved by the Ethics Committee on Animal Use (CEUA) of the University of Franca (CEUA/UNIFRAN, #033/15). To improve the quality of the clinical studies on ECT, we followed the design of Campana et al. [46]. 

This was a prospective, nonrandomized clinical study (2015–2017) using dogs admitted to the Veterinary Teaching Hospital of São Paulo State University and the Veterinary Teaching Hospital of the University of Franca with naturally occurring cutaneous SCCs that were treated with ECT. Eleven dogs were enrolled in the study, of which five had more than one lesion; thus, we investigated 18 lesions in 11 patients. Written consent to perform the treatment was obtained from the owner. The follow-up period was defined as the time from initial enrollment in the study until the last examination of the patient. The minimum follow-up period was 1 month. All dogs enrolled in this study fulfilled the following criteria: histopathologically confirmed diagnosis of SCC, absence of distant metastases, compliance of the owner with follow-up after 21 d, and the owner’s permission to perform biopsies before (D0) and after (D21) ECT treatment. The same patient group was used previously by our team to evaluate other parameters, and all available clinical and histological associations were evaluated previously [13].

Physical examination and biochemical evaluation (including complete blood count and blood urea nitrogen, creatinine, albumin, and alanine aminotransferase levels) were performed, along with thoracic radiography (three-view), abdominal ultrasound, and fine-needle aspiration of regional lymph nodes for tumoral staging according to the World Health Organization guidelines for skin tumors [47].

The electrochemotherapy protocol was based on a previously described technique [13] using bleomycin and doxorubicin, followed by a sequence of eight square-wave electrical pulses, each lasting 100 µs. The 1000 V/cm pulses, at a frequency of 1 Hz, were administered using a clinical electroporator (Electroporator LC BK-100, Brazil) fabricated in-house and were delivered using six needle electrodes with a 0.3 mm distance between them. The needle electrodes were arranged in rows (parallel array) until they completely covered the tumor. All ECT procedures were performed under general anesthesia. Day 21 was selected for further evaluation based on the schedule of doxorubicin treatment. Furthermore, we used the response evaluation criteria in solid tumors (RECIST) after ECT treatment and defined complete remission (CR) as the disappearance of all target lesions, partial remission (PR) as at least a 30% decrease in the sum of the diameters of the target lesions, progressive disease (PD) as at least a 20% increase in the sum of the diameters of the target lesions, and stable disease (SD) as neither sufficient shrinkage to qualify a PR nor a sufficient increase to qualify as PD [48].

Biopsies were obtained intratumorally before the first ECT session (D0) and on day 21 (D21) after ECT treatment. Areas with necrosis and macroscopic aspects of inflammation were avoided when selecting the tumor area. All samples were immediately placed in 10% formalin for 24 h, followed by 70% alcohol until the paraffin sections were prepared. Microscopic necrosis was classified as absent, grade I (+), grade II (++), or grade III (+++). MT staining was performed according to the method described by Prophet et al. [49], in which the intensity of collagen fiber staining was classified as mild (grade 1), moderate (grade 2), or accentuated (grade 3) and staining was classified as weak or strong. Intratumoral inflammatory infiltration was classified as lymphoplasmacytic, neutrophilic, or mixed (lymphocytes, plasma cells, neutrophils, and histiocytes) and the infiltration grade was classified as mild, moderate, or severe. These analyses were performed in an observational manner using a light microscope.

Immunohistochemical staining for VEGF and CD31 was performed for all SCC tissues using the original biopsy samples (D0) and the biopsy specimens collected 21 d after the first ECT session (D21), as previously described [50]. Immunohistochemical staining was performed using the peroxidase method and 3,3’-diaminobenzidine tetrachloride. The slides were dewaxed in xylene and rehydrated in a graded series of ethanol solutions. Citrate buffer (pH 6.0) was used for antigen retrieval in a pressure cooker (Pascal; Agilent Technologies, Santa Clara, CA, USA). Endogenous peroxidase was blocked with a commercial solution (Protein Block, Agilent Technologies), and the samples were incubated with the following primary antibodies overnight at 4 °C: monoclonal mouse anti-human VEGF121 isoform [51] (NeoMarkers, Fremont, CA, USA) at a 1:100 dilution and polyclonal rabbit anti-CD31 (ThermoFisher Scientific, Waltham, MA, USA) at a 1:50 dilution [52]. Immunohistochemical analysis was performed using an autostainer platform (Dako, Santa Clara, CA, USA). Harris hematoxylin was used for tissue counterstaining. Negative controls were included for all antibodies, using a universal negative control mouse antibody (Dako, Santa Clara, CA, USA) according to the manufacturer’s instructions. Positive control samples for all antibodies consisted of normal lymph nodes, according to the Protein Atlas guidelines (www.proteinatlas.org, accessed on 15 September 2021).

VEGF staining was classified with a score of 0 to 4, based on cytoplasmic immunostaining, as follows: 0, no staining; 1, 5–25% of cells immunostained; 2, 26%–50% of cells immunostained; 3, 51–75% of cells immunostained; and 4, >75% of cells immunostained. To evaluate CD31 levels, we analyzed five random high-power fields (400×), and the result was expressed as the median of the five fields for each sample.

For statistical analysis, we calculated the mean values of the histopathological and immunohistochemical parameters and classified the value for each parameter as “low” if it was less than the median or “high” if it was greater than the median.

The data are presented as the mean and standard deviation, and descriptive analyses were performed for the qualitative data. Fisher’s exact tests were used for all variables, when comparing groups D0 and D21. Statistical analyses were performed using Prism® 8.1.0 (GraphPad, San Diego, CA, USA) and *p* values < 0.05, were considered significant.

## 3. Results

Mixed-breed dogs (n = 5) were most found to be most commonly affected by SCC, followed by American pit bulls (n = 3), boxers (n = 2), and English pointers (n = 1). All patients had sparse fur and lightly pigmented skin. The mean age of the animals was 7.5 (standard deviation ± 2.29), with females being more common than males (n = 10). The most commonly affected sites were the abdominal region (n = 10), followed by the thoracic (n = 5), axillary (n = 1), preputial (n = 1), and tibial regions (n = 1). 

Based on the RECIST criteria, on day 21, of the 11 animals that underwent ECT, 45% presented with PR (5/11), 27% with PD (3/11), and 27% with SD (3/11). Of the 18 lesions that were treated, 61% (11/18) presented with PR, 22.2% with PD (4/18), and 16.6% with SD (3/18). The patients that did not present with CR on day 21 underwent another ECT session until CR or referral for surgery after cytoreduction. The number of sessions that each patient underwent ranged from one to three. Five patients needed a surgical procedure after follow-up due to tumoral macroscopic lesions or due to the owner’s request (Supplementary Table S1). At the end of the observational study, eight lesions responded to ECT and did not require surgery and all lesions presented with scar tissue (cutaneous fibrosis) and tissue retraction at the local site, with pinkish skin (Figure S1).

**Figure 1:**
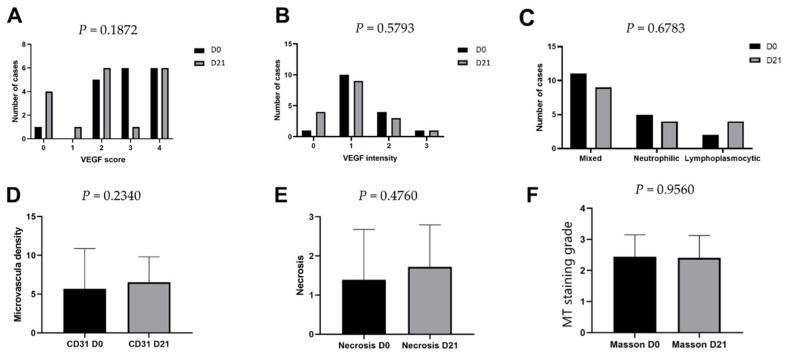
Expression levels of vascular endothelial growth factor and CD31, cellular infiltration, necrosis, and intratumoral collagen levels pre- and post-electrochemotherapy treatment in dogs with cutaneous squamous cell carcinoma. (**A**) Vascular endothelial growth factor (VEGF) staining score and (**B**) intensity pre-(day 0, D0) and post-(day 21, D21) electrochemotherapy (ECT) treatment. Subset of infiltrating cells pre- and post-ECT treatment (**C**). CD31 expression levels pre- and post-ECT treatment (**D**). Microscopic necrosis evaluation pre- and post-ECT treatment (**E**). Masson’s trichrome (MT) staining grade pre- and post-ECT treatment (**F**). No significant differences were observed in the parameters evaluated (*p* > 0.05).

**Figure 2:**
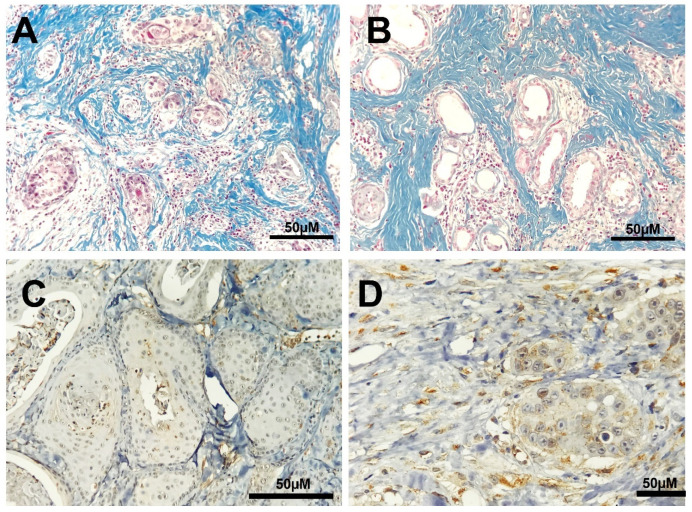
Photomicroscopy of cutaneous squamous cell carcinoma from dogs that underwent ECT. Accentuated staining, characterized by a greenish color (collagen fibers), is seen between the tumor tissue ((**A**,**B**); MT stain). Immunohistochemical staining of endothelial cells (CD31; (**C**)). Immunohistochemical staining showing cytoplasmic staining of VEGF (**D**).

VEGF expression was observed in 100% of cutaneous SCC lesions at both time points, with cytoplasmic staining in neoplastic cells and a granular pattern in endothelial cells. At D0, none of the lesions were classified as score 1 for VEGF staining, five lesions had a score of 2, six had a score of 3, and six had a score of 4. At D21, only 15 lesions were evaluated (three had inadequate tissue) with one sample had a score of 1, six lesions had a score of 2, one sample had a score of 3, and seven lesions had a score of 4. There was no significant difference in VEGF scores between D0 and D21 (*p* = 0.1872) or VEGF staining intensity during the evaluated period (*p* = 0.5793; Figure 1A,B and Figure 2C). The average scores for CD31 expression ranged from 0.8% to 14.8% at D0 and from 1.8% to 12.8% at D21, with no significant difference between the time points (*p* = 0.2340, Figure 1D and Figure 2D).

Mixed-pattern infiltration was the most common type of intratumoral cellular infiltration (n = 11), followed by neutrophilic (n = 5) and lymphoplasmacytic (n = 2) infiltration. On day 21, mixed cellular infiltration was the most common type observed, followed by equal amounts of neutrophilic (n = 4) and lymphoplasmacytic infiltration (n = 4). No significant difference in cellular infiltration was observed between the time points evaluated (*p* = 0.6783, Figure 1C). No significant difference was detected in the necrosis score 21 d after ECT (*p* = 0.476, Figure 1E).

Interestingly, regarding overall survival, the mixed-pattern infiltration was associated with better survival time (*p* = 0.0127) when compared to lymphoplasmacytic and neutrophilic pattern. No significant difference was detected for overall survival in the necrosis, MT, and VEGF score nor between well and poorly differentiated tumors (*p* > 0.05) (data not shown). 

MT staining revealed the presence of peritumoral connective tissue, which was mild in 2/18 (11.1%), moderate in 6/18 (33.3%), and accentuated in 10/18 (55.5%) samples before ECT. After the procedure, a similar distribution was observed in all samples (one sample was inadequate for analysis), with no differences observed in the staining of peritumoral connective tissue (*p* > 0.05; Table 1; Figure 1F and Figure 2A,B). 

## 4. Discussion

Most neoplasms release angiogenic factors, such as VEGF, which induce the growth of new blood vessels [53]. This process is related to tumor progression and metastasis [54]. We observed VEGF immunostaining in 100% of cutaneous SCCs. Similarly, previous studies have observed VEGF positivity ranging from 89% to 100% in SCCs in dogs [55,56].

Electroporation causes a reversible change in the cytoskeleton of endothelial cells, leading to edema, impairment of the endothelial barrier, and increased vascular resistance. This allows greater permeability of the vessel wall to macromolecules, and it decreases the rate of tumor growth. Due to the intimate contact between endothelial cells and the blood compartment, the endothelium tends to undergo greater electropermeabilization than the adjacent tissues. Consequently, endothelial cells are exposed to higher concentrations of antineoplastic drugs after their intravenous administration [57]. Thus, ECT induces endothelial apoptosis, causing blood flow obstruction, and subsequent ischemic death of all cells supplied by the blood vessel [58,59]. 

One possible mechanism underlying cell damage induced by ECT is ischemic injury. A previous study evaluated the in vivo responses of tumors and normal blood vessels to ECT, and detected no blood flow 24 h after the procedure, indicating a cytotoxic effect on tumor endothelial cells, leading to ischemic cell death. This finding indicated that ECT selectively acts on tumor blood vessels, leaving normal blood vessels intact [60,61]. 

In another study, two women were diagnosed with stage IIIc melanoma with multiple cutaneous metastases. Sequential histological and immunohistochemical analyses were performed before ECT and at 10 min, 3 h, 3 d, 10 d, 1 month, and 2 months after ECT. They observed that vasodilatation occurred because of acute inflammation and neoangiogenesis in the late phases. However, histological signs of vascular damage were scant and limited to endothelial cells and fibrin deposition in the 3- and 10-day specimens. Furthermore, they also observed initial fibrosis at 10 d, which increased in the superficial dermis after 1 month [62].

In this study, although ECT induced instantaneous changes in blood flow and the death of endothelial cells, it did not change the expression levels of the angiogenic markers, VEGF and CD31, on day D21. The time point used for evaluation after ECT may have been too late, as the microvascular alterations may have remained for up to 5 d after ECT [57,58]. The microvasculature may have been reestablished on the 21st day due to re-endothelization of preserved blood vessel scaffolds. Although neoangiogenesis may also occur in the late phase of the procedure, more time points should be included to understand the complexity of the microenvironment. One limitation of this study is the small number of samples obtained after the procedure. However, including animals from clinical practice to obtain sequential specimens is difficult because general anesthesia is needed for animals, as opposed to humans where cutaneous tumor nodules are usually obtained under local anesthesia. 

In addition, other markers, such as hypoxia-1 inducing factor 1 (HIF-1), may assist in understanding the effect of ECT on angiogenesis. HIF-1 affects transient hypoxia induced by ECT, as well as the tumor microenvironment after hypoxic stress, by inducing the expression of angiogenic factors, such as VEGF [59,63,64]. 

According to Spugnini et al. [40], it is possible to observe the presence of scar tissue and necrosis on day 14 post-ECT, with the occurrence of tissue rearrangements and tumoral remission in oral melanoma, soft tissue sarcoma in dogs and cats, mast cell tumors, and SCC. In this study, at the end of the treatment, eight lesions presented with scar tissue (cutaneous fibrosis) and tissue retraction at the local site, with pinkish skin, and this was consistent with the observations made by Spugnini et al. [40]. Although tumor remission has previously been observed due to a decrease in tumor volume [13], we found no change in the presence of fibroplasia in the tumor tissue, as assessed by MT, or an increase in necrosis. This may be expected as electroporation only affects the permeability of the cell membrane, without causing thermal ablation, as observed in a previous study [44], where the presence of an intact ECM was found to assist in tissue repair after the procedure. Necrosis is considered to be one of the local adverse effects of ECT, due to tumor remission. The extent of necrosis is related to the size of the tumor, and its presence is not related to pain or discomfort [65]. In the present study, no significant difference in necrosis was observed between day 0 and day 21, suggesting that necrosis may be a factor related to the tumor itself, as it was present before treatment. It is important to note that necrosis induced by ECT is usually more evident macroscopically in the first 7 d after treatment [65]; however, we observed necrosis on the 21st day by microscopic evaluation. Other studies have also observed the presence of necrosis 2–10 d after ECT [62,66].

Furthermore, we observed that most of the SCCs had a mixed pattern of cellular infiltration before the patients underwent ECT. After treatment, this pattern did not change in a late response (on day 21). This finding is in contrast to the findings of Spugnini et al. [40], who observed a significant change in the cellular pattern 14 d after ECT, with no inflammatory response and most of the tumor containing scar tissue. We also observed that patients had best overall survival for mixed-pattern infiltration when compared to other subtypes, suggesting that an heterogenous microenvironment may play a role in the response for local control. 

Although the incidence of cSCC in dogs is quite high in some countries, little is known about the inflammatory response in skin tumors, as most studies have focused on mammary tumors in dogs [25,26]. The importance of understanding the complex biology of cSCC, including the characterization of the inflammatory response, may benefit future studies and help to develop oncological immunotherapies. 

Previous studies have evaluated the inflammatory cell infiltration induced by ECT [67]. In a study of colorectal liver metastases, 8 weeks after ECT, a band of fibrous tissue and chronic inflammatory infiltrates, consisting of lymphocytes and plasma cells, were observed in the ablated area, while necrosis of blood vessels was observed at the site of electrode insertion [41]. This feature has also been observed in humans with cutaneous melanoma metastasis which show early signs of damage, including increased inflammatory infiltrate, 10 min after ECT. After 3 h, a massive mononuclear inflammatory infiltrate surrounds the tumor nodule. After 1 month, this inflammatory infiltrate is inverted, with a low level of mononuclear infiltration and a greater number of granzyme-B+ lymphocytes, suggesting that ECT helps with the long-term local tumor control by inducing persistent immune response of T-cytotoxic lymphocytes [62].

In another study on women with cutaneous melanoma metastasis, immunohistochemical analysis showed a striking prevalence of CD3+ T lymphocytes at all stages of tissue reaction after ECT. No CD20+ B cells were detected, but the infiltrate consisted largely of CD8+ cells (granzyme-B positive), with few CD4+ lymphocytes. This suggests a persistent immune response of T-cytotoxic lymphocytes, which may explain the long-term local tumor control by ECT. 

In contrast to the aforementioned studies, we did not observe a significant change in intratumoral cellular infiltration after ECT, with mixed-pattern infiltration being the most common type at both time points. Based on the results of previous studies, we expected a change in the inflammatory pattern, and the absence of an inflammatory component at day 21 post ECT. Instead, a massive inflammatory infiltrate was still present, suggesting that the skin of dogs may have a late-phase response during the course of remission. Another limitation of this study was that no samples were obtained from dogs in complete remission to determine the presence lymphocytic infiltrate, as observed in other studies [40,62].

Santana et al. [25] evaluated the inflammatory components in well-differentiated and poorly differentiated cSCCs in dogs. No significant difference was observed in the number of macrophages, T lymphocytes, or plasma cells between the different degrees of differentiation. A significant macrophage profile was observed in both groups; however, its role in the inflammatory infiltrate in SCC remain unclear. Further studies are needed to evaluate the different subpopulations of leukocytes in association with the inflammatory response in SCC. 

Although we did not perform immunohistochemical analyses to differentiate the subpopulation of leukocytes, lymphocytes were present at both time points, suggesting that either a protective response or tumor development was occurring. Previous studies have reported the presence of lymphocytes associated with tumor inflammatory infiltrates [68,69]. 

The combination of ECT plus immunotherapy, represented by immune checkpoint inhibitors, has been evaluated in humans with advanced cancer of the head and neck and non-melanoma skin cancer with potentially beneficial results [70,71]. This is important as ECT improves the local immune system and may stimulate the local infiltration of dendritic cells [72]. Although we did not observe a significant difference in cellular infiltration after ECT, lymphocytic infiltration tended to increase 21 d after therapy, suggesting local stimulation.

Our MT staining results were similar to those of another study showing 11.7% mild, 47% moderate, and 41% accentuated staining in dogs with cutaneous SCC [73]. In contrast, in human patients with prostate carcinoma, greater peritumoral stromal cell staining has been observed in those with a Gleason score of 6–7 (82.8%) than those with a Gleason score of 8–9 (15%) [74]. In addition, the authors of the previous study pointed out that stromal cells may shield cancer cells from the host immune response, and that a subset of tumors is functionally stroma-independent. Therefore, further studies are needed to determine whether the stromal compartment may preclude a favorable response to ECT. Although the aforementioned study associated the stromal component with histological grade (Gleason score), cell undifferentiation, and prognosis, it is important to note that our study did not involve survival analysis and only three dogs had undifferentiated carcinomas, with variable grades of MT staining. In our study, all lesions had a stromal component, even those that were undifferentiated, but this did not change significantly after treatment. 

One of the advantages of using MT staining is its low-cost and practical implementation compared to other techniques. It has been observed that higher intratumoral collagen levels are associated with lower-grade canine cutaneous mast cell tumors, with greater survival times in those with a value lower than 9.25% [29,32]. In female dogs with mammary tumors, aggressive carcinomas with shorter collagen fibers are present, which directly affects the survival time [34]. This has also been observed in feline mammary tumors, in which denser collagen, thicker and straighter fibers, and less-identifiable tumor-stromal boundaries are associated with poorer outcomes, independent of the clinical variables, thus increasing the predictive power of the clinical model [35].

MT staining was used to evaluate collagen distribution after ECT, with an expectation of increased collagen density. As tumor cells infiltrate the tumor stroma, ECT is expected to induce an antitumor response, a decreased number of neoplastic cells, and the replacement of the tumor cells with collagen. However, we did not observe any of these modifications before or after ECT.

Golberg et al. [75] evaluated skin rejuvenation after administration of a pulsed electric field (PEF). They observed prominent proliferation of the epidermis, microvasculature formation, and the secretion of collagen in treated areas, without scarring. They also observed a 43% increase in collagen synthesis, compared to the basal level, 3 weeks after PEF administration. Furthermore, the overexpression of p63, a regulator of keratinocyte proliferation and differentiation, was found to be correlated with increased levels of growth factors, such as IL-6, EGF, and VEGF. However, we did not observe an increase in angiogenesis or an induction of collagen synthesis 3 weeks after ECT, suggesting that the mechanisms of action may differ between dogs and rats.

Further research is needed to determine the angiogenic and stromal components in peritumoral and intratumoral areas and correlate these parameters with the response to electroporation-based treatment. Furthermore, we suggest that early and late post-ECT-treatment phases should be evaluated, as no changes were observed on day 21 in our study. In addition, the low number of samples in the present study obtained from clinical practice (18 lesions, 11 patients) limited further conclusions. A larger number of samples, with sequential histological and immunohistochemical analyses, are needed to understand the complexity of the response to ECT. 

## 5. Conclusions

ECT did not change VEGF levels, cellular infiltration, or intratumoral collagen proliferation in dogs with cutaneous SCC at the time point evaluated, suggesting that early and late post-ECT-treatment phases should be evaluated. Moreover, immunohistochemical characterization of the subpopulations of leucocytes should be performed to determine whether the cellular components influence the overall response. 

## Figures and Tables

**Table 1:** Number of samples according to Masson’s Trichrome staining intensity before and after electrochemotherapy treatment in dogs with cutaneous squamous cell carcinoma.

Intensity	Day 0	Day 21
Mild	2	2
Moderate	6	6
Accentuated	10	9

## Data Availability

Data is contained within the article or supplementary material.

## Supplementary Materials

The following are available online at https://www.mdpi.com/article/10.3390/life11121321/s1. Supplementary Figure S1: Patient ID2 diagnosed with cutaneous SCC. The lesion was observed macroscopically before and after ECT treatment and at complete remission on day 90. Supplementary Table S1: Expression of vascular endothelial growth factor (score and intensity), CD31 expression, cellular infiltration (subset and intensity), necrosis, and intratumoral collagen (grade and intensity) before (day 0, D0) and after ECT (day 21, D21) treatment in dogs with cutaneous squamous cell carcinoma.
