# Supplementary material for: VEGF Expression, Cellular Infiltration, and Intratumoral Collagen Levels after Electroporation-Based Treatment of Dogs with Cutaneous Squamous Cell Carcinoma
Source: Life (Basel). 2021 Nov 30;11(12):1321. doi: 10.3390/life11121321 (PMC8708059; doi:10.3390/life11121321)
Supplement: Supplementary file 1 [file life-11-01321-s001.zip › Supplementary Figure S1.pdf]

Supplementary Materials

Expression of vascular endothelial growth factor, cellular infiltration and evaluation of intratumoral collagen in dogs with cutaneous squamous cell carcinoma submitted to electroporation-based treatment

Dos Anjos et al.

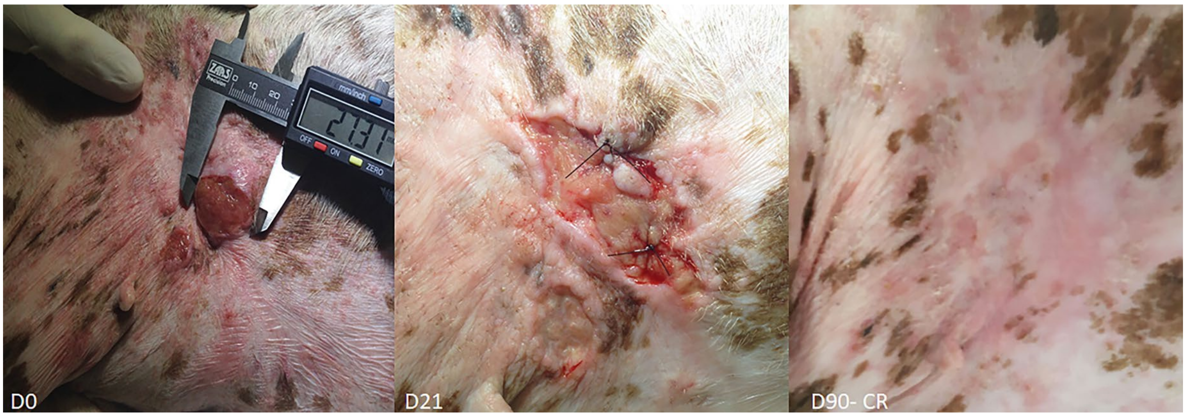

Supplementary Figure S1. Patient ID2 diagnosed with cutaneous SCC. It is possible to observe the macroscopic aspect of the lesion pre-, post-ECT treatment and in complete remission at day 90.
